# Development of a physical literacy assessment framework for Chinese preschool children: a Delphi–AHP approach

**DOI:** 10.3389/fpubh.2025.1650793

**Published:** 2025-08-19

**Authors:** Xiaojuan Tao, Junkai Zhang, Xiaotian Wang, Yuliu Tao, Mingming Guo

**Affiliations:** ^1^Physical Education and Sports School, Soochow University, Suzhou, Jiangsu, China; ^2^Department of Physical Education, College of Education for the Future, Beijing Normal University, Zhuhai, Guangdong, China

**Keywords:** physical literacy, preschool children, Delphi method, framework development, China

## Abstract

**Purpose:**

Physical literacy (PL) during early childhood is crucial for establishing a foundation for lifelong physical activity and holistic development. However, China currently lacks a developmentally appropriate and culturally relevant PL framework for preschool-aged children. This study aimed to develop such a framework using a modified Delphi method and determine the relative importance of its components through the Analytic Hierarchy Process (AHP).

**Method:**

The study employed a multi-phase design comprising a literature review, expert interviews, and two rounds of Delphi surveys with 40 experts across relevant fields. Items were retained if they met the 80% agreement threshold or were justified based on expert consensus and developmental relevance. The AHP was conducted using YAAHP software to calculate the relative weights of indicators at all levels.

**Results:**

The finalized framework comprises four core dimensions—motivation and confidence, physical competence, knowledge and understanding, and physical activity participation—encompassing 15 first-level and 49 second-level indicators. Dimensional weights were relatively balanced, with motivation and confidence (25.68%) and physical activity participation (25.52%) slightly exceeding physical competence (25.38%) and knowledge and understanding (23.42%).

**Conclusion:**

This study presents the first national PL framework specifically designed for Chinese preschoolers. It provides theoretical grounding and practical guidance for future PL assessments and early intervention strategies. The inclusion of risk prevention behaviors and 24-h movement behaviors—physical activity, sedentary behavior, and sleep—marks a significant advancement over existing models.

## 1 Introduction

Globally, the prevalence of health issues such as overweight, obesity, and motor development delays is increasingly observed at younger ages. Insufficient physical activity and prolonged screen time have been identified as major contributing factors to this trend (1). Studies have shown that early intervention during childhood can effectively alter behavior and reduce the likelihood of overweight and obesity in adulthood (2). In this context, “physical literacy (PL)” has emerged as a novel strategy for addressing the early onset of non-communicable diseases and has become a growing focus in international physical education research (3).

PL spans the entire life course, and the preschool stage is recognized by Margaret Whitehead as the starting point of the “journey of PL” (4). This period is not only critical for the development of fundamental movement skills but also plays a vital role in establishing healthy behaviors, enhancing social and emotional wellbeing, and fostering cognitive skills. Therefore, interventions aimed at improving PL in preschool children are essential for unlocking their movement potential and supporting holistic development. Providing opportunities for PL development at this stage is crucial for ensuring a strong start and long-term success in life (5).

Although international interest in PL has grown, research focusing on preschool-aged children remains scarce and is largely confined to government or NGO reports from developed countries such as the United Kingdom, Canada, the United States, and Australia (6). Given this limitation, there is a growing need for contemporary studies to move beyond the traditional emphasis on physical activity and health outcomes, and instead address all dimensions of PL in a more holistic manner (7). As childhood health issues—such as obesity, mental health disorders, cardiovascular disease, and diabetes—become increasingly prevalent, clarifying the definition of PL and identifying its core components has emerged as a critical global research priority.

As the country with the second largest child population globally (8), China faces significant issues such as insufficient physical activity (9), an alarming trend of early-onset health risks (10), and delayed development of fundamental motor skills (11) among preschoolers. Thus, promoting PL in Chinese preschool children is of utmost importance. However, key questions remain unanswered: What are the components of PL for this age group? How can PL be developed systematically? Which dimensions should be targeted during sensitive developmental periods?

Currently, understanding of the “PL journey” in China is limited, and most existing research focuses on school-aged children in stable environments (7). There is a lack of empirical studies on PL interventions in preschoolers, primarily due to the absence of a clearly defined framework for this age group. As a result, the development and implementation of PL interventions have lagged behind those in developed countries.

Against this backdrop, the present study aims to localize and define the concept of PL for Chinese preschool children using a modified Delphi approach and to establish a comprehensive, developmentally appropriate PL framework tailored to China's sociocultural context. Specifically, the study seeks to identify the core components of PL relevant to this age group, determine how these components can be systematically developed through expert consensus, and highlight the dimensions that should be prioritized during sensitive developmental periods. By addressing these objectives, the study responds directly to the urgent need for a clear and context-specific foundation for early childhood PL assessment and promotion in China.

## 2 Method

### 2.1 Study design

This study developed a content framework for PL tailored to Chinese preschool children through a series of systematic steps. First, a preliminary framework was established based on a review of relevant literature and expert telephone interviews. These sources helped identify key developmental characteristics, environmental contexts (e.g., preschool and community settings), and health-related needs (e.g., physical fitness, motor development) of Chinese preschoolers, which were integrated into the initial indicator pool to ensure contextual relevance.

Subsequently, a modified Delphi process was employed to screen and refine the framework's components, first-level indicators, and second-level indicators through two iterative rounds. The experts consulted during the telephone interview stage were the same as those who participated in the Delphi surveys. Finally, the Analytic Hierarchy Process (AHP) was used to calculate the relative importance of each indicator.

### 2.2 Literature search

The literature review aimed to construct a theoretical structure and an initial pool of indicators for the PL framework for Chinese preschoolers. Studies published in English or Chinese that addressed the concept, characteristics, or framework of PL were included. Literature was retrieved from four electronic databases: CNKI, EBSCO, Web of Science Core Collection, and ProQuest. English-language literature was searched from March 2001 to December 2024, while Chinese-language literature was searched from March 2012 to December 2024. Keywords used included “Physical Literacy,” “Physical Literacy for Preschoolers,” “Physical Literacy for Early Childhood,” and “Physical Literacy System for Preschoolers.”

### 2.3 Expert selection

Following the expert selection strategy employed by Zhang et al. (12), experts were identified through systematic searches of core domestic and international academic journals, official websites of relevant international organizations, and peer recommendations. Eligibility criteria required experts to be professors or researchers based in China, engaged in studies related to PL, physical activity promotion, or sport psychology, and with a demonstrated interest in PL.

Given that preschool children spend significant time with their parents, and that parents play an irreplaceable role in fostering children's PL, parents were also included in the expert panel. In total, 40 experts representing diverse academic disciplines relevant to the study were invited via email or WeChat and agreed to participate in the Delphi consultation. All 40 experts completed the first and second rounds of the Delphi survey. Table 1 summarizes the demographic characteristics of the experts, with the full list provided in Appendix Table 1.

**Table 1:** Characteristics of the experts (*n* = 40).

**Demographic**	**Category**	***n* (%) / Mean**
Gender	Male	24 (60%)
	Female	16 (40%)
Years of work^a^	Average	16.9
	Range	1–49
Education background	Bachelor	12 (30%)
	Master	8 (20%)
	PhD	20 (50%)
Professional job title^b^	Professor	13 (32.5%)
	Associate professor	17 (42.5%)
	Assistant professor	3 (7.5%)
	Teaching assistant	7 (17.5%)
Area of expertise^c^	School physical education	9 (22%)
	Physical literacy	7 (18%)
	Preschool physical education	7 (18%)
	Preschool instruction	7 (18%)
	Preschool childcare	5 (12%)
	Preschool education	4 (10%)
	Philosophy of physical education	1 (2%)

^a^Years of work were calculated as of September 1, 2024.

^b^All professional job titles were standardized based on the commonly used academic rank system in China: Teaching Assistant, Assistant Professor, Associate Professor, and Professor.

^c^In the “Area of Expertise” category, “Preschool Education” refers to scholars focused on preschool education theory; “Preschool Physical Education” refers to experts in physical education working in early childhood contexts; and “Preschool Instruction” refers to frontline practitioners such as preschool PE teachers or coaches at early childhood sports clubs.

Notably, the years of work experience were calculated as of September 1, 2024—prior to the formal Delphi rounds (April–May 2025)—as this was the point at which expert background information was obtained during the initial recruitment phase. This approach ensured consistency across all experts.

### 2.4 Telephone interview

As a well-established method, telephone interviews have been widely used in the field of sociology (13, 14), and are frequently employed in Delphi studies (12). Therefore, prior to launching the Delphi survey, we conducted telephone interviews with the selected panel of experts. The purpose of the interviews was to gather expert opinions on the importance, comprehensiveness, and relevance of a proposed Early Childhood Physical Literacy System, and to revise the initial theoretical structure and indicator pool accordingly. These interviews took place between February and March 2025. Based on expert feedback, the initial indicator pool was refined to better reflect the structural components, first-level indicators, and second-level indicators of PL in Chinese preschool children. This revised version was used to draft the first-round Delphi questionnaire.

### 2.5 Delphi study

The Delphi method is a research approach particularly well-suited for addressing real-world problems where expert input is essential (15). In this study, a modified Delphi technique was implemented entirely online, using an iterative mixed-methods design to reach expert consensus. To reduce potential bias—particularly social desirability bias—and to encourage independent judgment, all responses were anonymized. After each round, the research team synthesized both quantitative ratings and qualitative feedback from the experts and revised the questionnaire accordingly. A comprehensive summary, incorporating consensus statistics and thematically coded qualitative comments, was provided to each expert to support informed re-evaluation. All experts provided electronic informed consent prior to participation.

### 2.6 Two-round Delphi survey

Two Delphi rounds were conducted to balance the pursuit of expert consensus with the risk of participant fatigue and declining response rates—common challenges in Delphi studies involving multiple rounds (15, 16). Prior research suggests that two rounds are typically sufficient to stabilize expert opinions, particularly when the initial framework is grounded in empirical evidence and informed by preliminary interviews (12).

The Delphi process was conducted online between April and May 2025. The first-round questionnaire was developed based on a comprehensive literature review and insights from preliminary expert interviews. In Round 1, 40 experts were invited to complete the survey within 2 weeks, with reminders sent after 10 days if needed. Experts were asked to evaluate the framework's components, first-level indicators, and second-level indicators using a 3-point Likert scale (1 = Not appropriate, 2 = Needs revision, 3 = Appropriate), and to provide open-ended suggestions for modification, retention, or deletion. A 3-point scale was chosen to ensure clarity of judgment, reduce cognitive load on respondents, and enhance response efficiency across experts with varied disciplinary backgrounds. This simplified scale has also been widely used in prior Delphi studies aiming to establish consensus on content frameworks (17).

Based on first-round results, the research team performed a thematic analysis of the qualitative feedback from open-ended responses and integrated this with the consensus rates to develop the second-round questionnaire. This ensured that iteration decisions were guided not only by statistical thresholds but also by expert reasoning and contextual insight.

Before being invited to complete the second-round questionnaire, experts received a detailed summary of the first-round results, including specific feedback from other panelists on each indicator. This information not only facilitated the re-evaluation of revised items but also encouraged experts to reflect on the collective insights of the group.

### 2.7 Consensus

To determine expert consensus across the two Delphi rounds, different criteria were applied at each stage. In the first round, consensus was defined as 80% or more of the experts rating an item as either “2” (needs revision) or “3” (appropriate), following commonly accepted standards in Delphi research (15). Items meeting this threshold were retained without modification, while those falling below were further reviewed based on qualitative feedback and theoretical relevance. In the second round, a more rigorous statistical approach was adopted, incorporating three criteria: (1) the mean importance score (M) was greater than or equal to M – 2SD, (2) the full-score frequency (FF) was greater than or equal to FF – 2SD, and (3) the coefficient of variation (CV) was less than or equal to CV + 2SD. Items that satisfied all three criteria were retained. In a few cases where an item narrowly missed one criterion—particularly the CV threshold—it was still retained if supported by strong qualitative feedback and deemed theoretically important by the research team. This two-stage consensus process ensured both methodological rigor and content validity in the final framework.

### 2.8 Analytic hierarchy process

To determine the relative importance of each element within the PL framework, the AHP was applied following the completion of the second round of the Delphi survey. Experts were asked to perform pairwise comparisons using structured judgment matrices at three hierarchical levels: (1) components, (2) first-level indicators under each component, and (3) second-level indicators under each first-level indicator.

A 1–9 Saaty scale was used for all pairwise comparisons to quantify expert judgments. Each comparison assessed the relative importance of two elements with respect to a higher-level criterion. These pairwise judgments were used to construct reciprocal matrices, which formed the basis for calculating the relative weights of components, first-level indicators, and second-level.

To ensure the logical consistency of expert evaluations, a consistency ratio (CR) was calculated for each matrix. A CR value of 0.1 or lower was considered acceptable. Responses with CR values exceeding this threshold were reviewed for inconsistency and either clarified with the expert or excluded from the final analysis.

The aggregated group judgment for each matrix was derived using the arithmetic mean method. All AHP computations, including the construction of judgment matrices, consistency ratio calculations, and final weight derivation, were performed using YAAHP software (Version 12.6).

### 2.9 Data analysis

Descriptive statistics for expert characteristics (e.g., frequencies, percentages, means, and standard deviations), consensus rates, and item rating distributions were analyzed using IBM SPSS Version 27.0.

The AHP was employed to determine the specific weights of each component, first-level, and second-level indicator in the PL framework. Expert pairwise comparison data were analyzed using YAAHP software (Yuanjuece Software and Technology Ltd., Taiyuan, China; Version 12.6).

## 3 Results

### 3.1 Theoretical structural model and components of physical literacy in preschoolers

Before constructing the content framework for PL, it was necessary to clarify its conceptual definition. Based on a review of existing literature on PL, and through distinguishing it from related concepts such as health literacy and sports literacy, this study defines PL in the preschool stage (ages 3–6) as:

“*A comprehensive disposition developed through physical activity participation—primarily via fundamental motor skills—that enables children to begin to acquire motivation and confidence, physical competence, knowledge and understanding, and the ability to engage in physical activities for health challenges both now and in the future.”*

**Figure 1:**
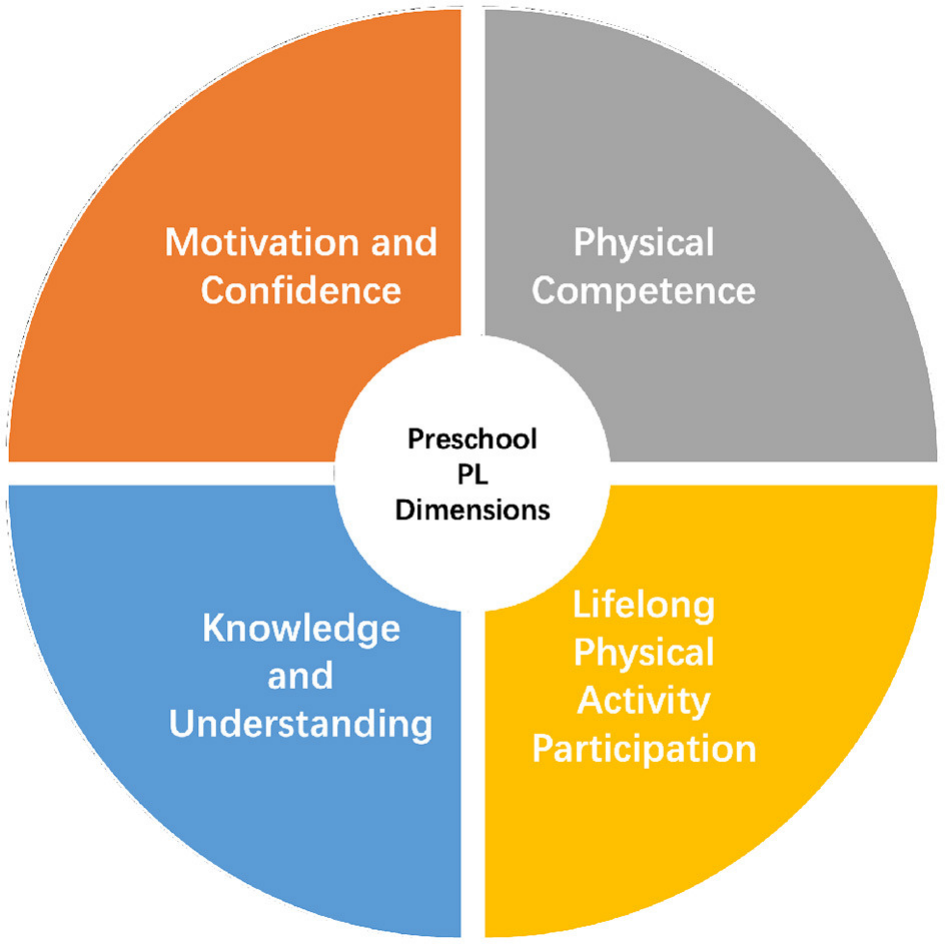
Four core components of preschool physical literacy.

Following an integrative review of both domestic and international research on PL and early childhood PL, this study identified four key dimensions of preschool PL: motivation and confidence, physical competence, knowledge and understanding, and lifelong physical activity participation (see Figure 1). These dimensions correspond respectively to the affective, physical, cognitive, and behavioral domains.

Analysis of the expert interview data revealed that 90% of experts agreed with the initial three dimensions, confirming their theoretical soundness. However, only 65% supported the inclusion of the fourth dimension, “lifelong physical activity participation,” indicating a need to revise its formulation to better suit the developmental characteristics of preschool children.

### 3.2 Development of an initial physical literacy framework

Referring to key national documents such as the Guidelines for Learning and Development of Children Aged 3–6 (18), and based on the four dimensional indicators, the study developed a draft framework through inductive and deductive methods. The framework included four dimensional indicators, 16 first-level indicators, and 48 second-level indicators.

These indicators span the three learning levels commonly used in preschool education in China (junior, middle, and senior classes). Table 2 presents the full preliminary indicator pool.

**Table 2:** Preliminary preschool physical literacy framework^a^.

**Domain**	**Sub-domain**	**Element**
1. Motivation and confidence	1.1 Curiosity and interest in physical activity	1.1.1 Shows curiosity, novelty-seeking and enthusiasm for physical games/activities; tries and participates proactively.
		1.1.2 Interested in the use of equipment and facilities.
		1.1.3 Has favorite sports/games and is committed to 1–2 regularly.
		1.1.4 Appreciates specific sports, follows events or athletes, cheers for success.
	1.2 Motivation for physical activity	1.2.1 Has intrinsic drive to participate actively in physical games/activities.
		1.2.2 Gets extrinsic motivation from environment to participate.
	1.3 Confidence in physical activity	1.3.1 Holds a winning belief in completing tasks.
		1.3.2 Can overcome fear and anxiety in physical challenges.
		1.3.3 Enjoys pleasure and satisfaction after completion.
2. Physical competence	2.1 Fundamental movement skills	2.1.1 Masters locomotor fundamental movement skills.
		2.1.2 Mastery of ball skills: throwing, catching, dribbling, etc.
		2.1.3 Mastery of non-locomotor skills: balancing, rolling, etc.
	2.2 Physical coordination	2.2.1 Displays agility and rhythm with music during activity.
		2.2.2 Demonstrates core body stability in motion.
		2.2.3 Shows muscular coordination in explosive tasks.
	2.3 Health-related fitness	2.3.1 Demonstrates cardiorespiratory endurance.
		2.3.2 Shows muscular strength through duration of activity.
		2.3.3 Possesses flexibility in shoulders, hips, and trunk.
	2.4 Environmental adaptability and risk-taking	2.4.1 Adapts to outdoor environments and varied conditions.
		2.4.2 Uses available equipment for diverse outdoor activities.
		2.4.3 Willingly engages in adventurous physical tasks.
3. Knowledge and understanding	3.1 Knowledge of physical activity	3.1.1 Knows basic body structure & own status.
		3.1.2 Knows pronunciation & writing of movement vocab.
		3.1.3 Knows the benefits of physical activity.
		3.1.4 Knows how to perform physical activity safely.
		3.1.5 Aware of appropriate timing for physical activity.
	3.2 Perceptual-motor competence	3.2.1 Sensitive to own bodily reactions during activity.
		3.2.2 Accurately judges personal motor and activity limits.
		3.2.3 Recognizes discomfort signs like cramps or pain.
	3.3 Role awareness in games	3.3.1 Understands and imitates social roles in games.
		3.3.2 Aware of role responsibilities in group play.
	3.4 Safety and self-protection	3.4.1 Practices basic injury prevention strategies.
		3.4.2 Identifies and avoids hazards in the environment.
		3.4.3 Participates cautiously; stops when unwell.
	3.5 Understanding of moral principles	3.5.1 Has basic rule awareness.
		3.5.2 Follows basic social rules in daily life.
		3.5.3 Alerts others to danger during activity
		3.5.4 Avoids harmful acts during activity
4. Lifelong physical activity participation	4.1 Individual responsibility	4.1.1 Views physical activity as personal responsibility.
		4.1.2 Shows perseverance and courage.
		4.1.3 Contributes for collective honor.
	4.2 Lifelong healthy lifestyle	4.2.1 Forms proactive exercise habit (60 min + 60 min).
		4.2.2 Maintains good sleep habits and rest.
		4.2.3 Forms healthy dietary habits.
	4.3 Prosocial behaviors	4.3.1 Communicates and shares during physical activity.
		4.3.2 Helps and comforts peers in difficulty.
	4.4 Participation in emerging activities	4.4.1 Participates in novel and evolving physical games.
		4.4.2 Innovates games using existing resources.

^a^This table presents the preliminary framework developed before the first round of the Delphi survey.

### 3.3 First round of Delphi survey results

In the first round of the Delphi process, questionnaires were distributed to 40 experts, and 37 valid responses were received. Feedback regarding the four dimensions, 16 first-level indicators, and 48 second-level indicators—as well as corresponding revisions—is summarized in Table 3.

**Table 3:** Summary of round 1 expert ratings on each indicator^a^.

**Domain**	**Sub-domain**	**Element**	**Keep (*n*)**	**Keep (%)**	**Discussion result**
1			37	100.0	Keep
	1.1		36	97.3	Keep
		1.1.1	37	100.0	Suggested revision
		1.1.2	35	94.6	Suggested revision
		1.1.3	34	91.9	Suggested revision
		1.1.4	32	86.5	Suggested revision
	1.2		36	97.3	Keep
		1.2.1	34	91.9	Suggested revision
		1.2.2	35	94.6	Suggested revision
	1.3		36	97.3	Keep
		1.3.1	35	94.6	Suggested revision
		1.3.2	36	97.3	Suggested revision
		1.3.3	36	97.3	Suggested revision
2			37	100.0	Keep
	2.1		37	100.0	Keep
		2.1.1	36	97.3	Suggested deletion
		2.1.2	36	97.3	Keep
		2.1.3	35	94.6	Keep
	2.2		32	86.5	Suggested deletion
		2.2.1	24	64.9	Suggested deletion
		2.2.2	24	64.9	Suggested deletion
		2.2.3	24	64.9	Suggested deletion
	2.3		36	97.3	Keep
		2.3.1	32	86.5	Suggested revision
		2.3.2	32	86.5	Suggested revision
		2.3.3	34	91.9	Suggested revision
	2.4		35	94.6	Suggested revision
		2.4.1	32	86.5	Suggested revision
		2.4.2	35	94.6	Suggested revision
		2.4.3	31	83.8	Suggested revision
3			37	100.0	Keep
	3.1		36	97.3	Suggested revision
		3.1.1	31	83.8	Suggested revision
		3.1.2	34	91.9	Suggested revision
		3.1.3	28	75.7	Suggested revision
		3.1.4	30	81.1	Suggested deletion
		3.1.5	32	86.5	Suggested revision
	3.2	3.2.1	33	89.	Keep
			34	91.9	Suggested re-assigned & modified.
		3.2.1	34	91.9	Suggested revision
		3.2.3	33	89.2	Suggested revision
	3.3		33	89.2	Keep
		3.3.1	35	94.6	Suggested revision
		3.3.2	36	97.3	Keep
	3.4		34	91.9	Keep
		3.4.1	32	86.5	Suggested revision
		3.4.2	35	94.6	Suggested revision
		3.4.3	35	94.6	Suggested revision
	3.5		29	78.4	Suggested re-assigned
		3.5.1	36	97.3	Suggested re-assigned
		3.5.2	33	89.2	Suggested deletion
		3.5.3	37	100.0	Suggested re-assigned
		3.5.4	37	100.0	Suggested re-assigned
4	4.1		35	94.6	Suggested re-assigned
			28	75.7	Retained based on subjective judgment.
		4.1.1	31	83.8	Suggested revision
		4.1.2	31	83.8	Suggested revision
		4.1.3	34	91.9	Suggested revision
	4.2		26	70.3	Retained based on subjective judgment.
		4.2.1	33	89.2	Suggested revision
		4.2.2	33	89.2	Suggested revision
		4.2.3	33	89.2	Suggested revision
	4.3		28	75.7	Retained based on subjective judgment.
		4.3.1	36	97.3	Keep
		4.3.2	37	100.0	Suggested revision
	4.4		29	78.4	Suggested deletion
		4.4.1	34	91.9	Suggested deletion
		4.4.2	33	89.2	Suggested deletion

^a^The contents corresponding to each serial number in this table are detailed in Table 2.

For the four dimensional indicators, expert consensus rates were high. The first three dimensions—motivation and confidence, physical competence, and knowledge and understanding—each received a 100% consensus rate. The fourth dimension, lifelong physical activity participation, received a 94.6% consensus rate. Some experts believed this dimension did not align well with the developmental characteristics of preschool children and suggested that emphasizing “lifelong” may set overly high expectations for this age group. Therefore, based on expert feedback, the dimension was retained but revised from “lifelong physical activity participation” to simply “physical activity participation.”

Regarding the 16 first-level indicators, most expert concerns focused on the developmental appropriateness of the indicators for children aged 3–6. Specific concerns included whether the indicators were too advanced, whether the vertical logic between dimension and first-level indicators was coherent, and whether the lateral relationships among indicators were clearly defined. Based on these concerns, the research team revised each indicator individually. Experts also proposed adding new indicators. After reviewing key policy documents such as the Guidelines for Learning and Development of Children Aged 3–6, a new first-level indicator was added: “2.1 Posture.” Meanwhile, two were removed: “2.2 Physical Coordination,” and “4.4 Participation in Emerging Activities.” One indicator was reassigned to another dimension: “3.5 Understanding of Moral Principles.” Two indicators—“2.4 Environmental Adaptability and Risk-taking” and “4.2 Lifelong Healthy Lifestyle”—were revised for clarity and alignment with child development principles. Although four indicators (4.1, 4.2, 4.3, and 4.4) did not meet the 80% consensus threshold, they were retained based on expert open-ended feedback highlighting their foundational relevance to preschoolers' social-emotional development. The research team carefully reviewed these suggestions and determined that their conceptual importance justified retention, while minor wording refinements were made to address expert concerns.

For the 48 second-level indicators, expert feedback centered on internal logic, consistency with parent indicators, and clarity of expression. Many frontline practitioners suggested making the indicators more concrete to enhance their practical applicability in teaching. Accordingly, the research team carefully refined each indicator for clarity and relevance. Specifically, 8 new second-level indicators were added (e.g., “1.3.1 Able to shift attention and emotions during physical play and maintain emotional stability”), 37 indicators were revised for clarity and alignment (e.g., “1.1.1 Holds sufficient curiosity and enthusiasm for physical activity”), and 2 indicators were removed due to redundancy or lack of clarity (see Appendix Table 2).

Table 3 presents a statistical summary of expert feedback on the indicators following the first round.

### 3.4 Second round of Delphi survey results

In the second round of the Delphi process, the revised version of the preschool PL framework (Version 2.0) was distributed to the 37 participating experts, and all completed the survey. Expert feedback on the 4 dimensional indicators, 15 first-level indicators, and 48 second-level indicators is summarized in Table 4.

**Table 4:** Summary of results from the second round of expert consultation^a^.

**No**.	**M**	**SD**	**FF**	**CV**	**M ≥M – 2 × SD**	**FF ≥FF – 2 × SD**	**CV ≤ CV + 2 × SD**
1	3	0	1.00	0	3	1	0
2	2.97	0.16	0.97	0.05	2.64	0.65	0.38
3	2.92	0.36	0.94	0.12	2.2	0.22	0.84
4	2.82	0.46	0.84	0.16	1.9	−0.07	1.08
1.1	2.97	0.16	0.97	0.05	2.64	0.65	0.38
1.2	2.94	0.23	0.95	0.07	2.49	0.49	0.53
1.3	2.97	0.16	0.97	0.05	2.65	0.65	0.38
2.1	2.82	0.56	0.89	0.2	1.69	−0.23	1.32
2.2	2.97	0.16	0.97	0.05	2.65	0.64	0.38
2.3	2.89	0.39	0.92	0.13	2.11	0.14	0.91
2.4	2.92	0.36	0.95	0.12	2.2	0.23	0.84
3.1	2.79	0.58	0.86	0.21	1.63	−0.29	1.36
3.2	2.92	0.36	0.95	0.12	2.2	0.23	0.84
3.3	2.71	0.61	0.78	0.22	1.49	−0.44	1.45
3.4	2.87	0.47	0.92	0.16	1.92	−0.03	1.12
4.1	2.82	0.56	0.89	0.19	1.63	−0.23	1.32
4.2	2.95	0.23	0.95	0.22	2.49	0.49	0.53
4.3	2.76	0.63	0.86	0.22	1.49	−0.41	1.5
4.4	2.71	0.61	0.81	0.22	1.49	−0.41	1.45
1.1.1	2.92	0.27	0.92	0.09	2.37	0.37	0.64
1.1.2	2.87	0.47	0.92	0.16	1.92	−0.03	1.12
1.1.3	2.87	0.41	0.89	0.14	2.04	0.06	0.97
1.1.4	2.84	0.41	0.89	0.17	1.85	−0.10	1.16
1.2.1	2.95	0.49	0.95	0.07	2.49	0.49	0.53
1.2.2	2.92	0.22	0.92	0.09	2.37	0.37	0.64
1.3.1	2.76	0.59	0.84	0.21	1.58	−0.34	1.39
1.3.2	2.92	0.27	0.92	0.09	2.37	0.37	0.64
1.3.3	2.89	0.31	0.89	0.1	2.27	0.27	0.73
1.3.4	2.87	0.34	0.86	0.11	2.18	0.17	0.8
2.1.1	2.92	0.36	0.95	0.12	2.20	0.23	0.84
2.1.2	2.76	0.63	0.86	0.22	1.49	−0.40	1.5
2.2.1	2.84	0.37	0.84	0.13	2.10	0.10	0.87
2.2.2	2.79	0.16	0.97	0.05	−0.32	0.65	0.85
2.2.3	3	0.00	1.00	0	3.00	1.00	0
2.3.1	2.82	0.46	0.84	0.16	1.90	−0.07	1.08
2.3.2	2.89	0.39	0.92	0.13	2.12	0.14	0.91
2.3.3	2.82	0.46	0.81	0.16	1.90	−0.10	1.08
2.3.4	2.95	0.32	0.97	0.11	2.30	0.32	0.76
2.4.1	2.82	0.46	0.84	0.16	1.90	−0.07	1.08
2.4.2	2.95	0.32	0.97	0.11	2.30	0.32	0.76
2.4.3	2.92	0.36	0.95	0.12	2.20	0.23	0.84
2.4.4	3	0.00	1.00	0	3.00	1.00	0
3.1.1	2.95	0.23	0.97	0.07	2.50	0.52	0.53
3.1.2	2.79	0.53	0.84	0.18	1.73	−0.22	1.25
3.1.3	2.89	0.31	0.89	0.1	2.27	0.27	0.73
3.1.4	2.92	0.35	0.95	0.12	2.20	0.23	0.84
3.1.5	2.82	0.46	0.84	0.16	1.90	−0.07	1.08
3.2.1	2.95	0.23	0.95	0.07	2.49	0.50	0.53
3.2.2	2.87	0.47	0.92	0.16	1.91	−0.03	1.11
3.3.1	2.92	0.36	0.95	0.12	2.20	0.23	0.84
3.3.2	2.89	0.45	0.95	0.15	1.99	0.04	1.06
3.4.1	2.89	0.39	0.92	0.13	2.11	0.14	0.91
3.4.2	2.84	0.44	0.86	0.15	1.97	−0.01	1.02
3.4.3	2.92	0.27	0.92	0.09	2.37	0.37	0.64
3.4.4	2.95	0.23	0.95	0.07	2.49	0.50	0.53
4.1.1	2.92	0.36	0.95	0.12	2.20	0.23	0.84
4.1.2	2.95	0.23	0.95	0.07	2.49	0.50	0.53
4.1.3	2.87	0.47	0.92	0.16	1.92	−0.03	1.11
4.2.1	2.97	0.16	0.97	0.05	2.64	0.65	0.38
4.2.2	2.87	0.41	0.89	0.14	2.04	0.06	0.97
4.2.3	2.89	0.45	0.95	0.15	1.99	0.04	1.06
4.2.4	2.89	0.45	0.95	0.15	1.99	0.04	1.06
4.3.1	2.92	0.36	0.95	0.12	2.20	0.23	0.84
4.3.2	2.89	0.39	0.92	0.13	2.12	0.14	0.91
4.3.3	2.89	0.45	0.95	0.15	1.99	0.04	1.06
4.4.1	2.89	0.45	0.95	0.15	1.99	0.04	1.06
4.4.2	2.87	0.47	0.92	0.16	1.92	−0.03	1.12
4.4.3	2.82	0.56	0.89	0.19	1.69	−0.24	1.32

^a^The contents corresponding to each serial number in this table are detailed in Table 2.

For the dimensional indicators, the mean importance ratings (M) for all four dimensional indicators were above the threshold of 2.5, indicating a high level of expert agreement on their relevance. Specifically, the dimension of Motivation and Confidence received the highest rating (*M* = 3.00), followed closely by Physical Competence (*M* = 2.97), Knowledge and Understanding (*M* = 2.92), and Physical Activity Participation (*M* = 2.82).

These results demonstrate a high level of consensus among experts regarding the importance of each dimension. The coefficients of variation (CV) for all dimensions were below 0.25, indicating low variability in expert evaluations. Moreover, the full-score frequency (FF) for each indicator exceeded 30%. All three predefined screening criteria were met: (1) the mean importance score (M) was greater than or equal to M – 2SD, (2) the full-score frequency (FF) was greater than or equal to FF – 2SD, and (3) the coefficient of variation (CV) was less than or equal to CV + 2^*^SD.

Qualitative feedback also supported these findings. While a few experts suggested replacing the term “Physical Activity Participation” with “Sports Participation,” the research team determined that the former was more comprehensive. For instance, watching sports events is a form of PL expression but may not be classified as “sports participation” in a narrow sense. Thus, “Physical Activity Participation” was retained as the final term. Based on both the quantitative and qualitative analysis, all four dimensions were included in the final framework.

For the first-level indicators, all 15 indicators achieved mean importance scores above 2.5 (range: 2.71 to 2.97), with full-score frequencies between 71% and 97%. The coefficients of variation were all below 0.25, indicating high consistency among experts. All three screening criteria were met. Additionally, several experts recommended translating the term Perceived Motor Competence to Motor Perception Ability to better align with common terminology used in Chinese sports science literature. This revision was adopted.

For the second-level indicators, all 48 second-level indicators met the three predefined inclusion criteria, indicating strong expert agreement on their importance. Specifically, the mean importance scores (M) for all items exceeded 2.5, ranging from 2.71 to 3.00; the coefficients of variation (CV) were all below 0.25, reflecting low variability; and the full-score frequencies (FF) for all items were above 30%, demonstrating broad expert support.

However, two items—“1.1.4 Watches sports events or programs with peers or adults” and “2.1.2 Maintaining correct posture with adult reminders”—showed outlier values in the third screening criterion (CV > CV + 2^*^SD). Qualitative feedback indicated that experts were concerned about the appropriateness of screen-based behaviors in the first item, given concerns about eye health. Others questioned whether preschool children were genuinely engaged by televised sports. Drawing on prior observational studies of children watching the Beijing Olympics with adult guidance, the research team revised the item to:

“*1.1.4 Can watch sports events or related TV programs for about 30 minutes with peers or adults and shows interest in physical activities.”*

For “2.1.2,” experts suggested replacing “adults” with “others” to reflect the potential role of peers in posture correction. The team also verified that posture maintenance is explicitly addressed in the Guidelines for Learning and Development of Children Aged 3–6. Therefore, the indicator was revised to:

“*2.1.2 Can maintain correct standing, sitting, and walking postures when reminded by others.”*

Following the analysis and revisions from the second round, the final preschool PL framework was established, consisting of four dimensional indicators, 15 first-level indicators, and 49 second-level indicators. A complete summary of the statistical results for the second round is presented in Table 4.

### 3.5 Weights for indicators of the preschool physical literacy framework

Results from the AHP revealed the following weight distribution across the four core dimensions of the preschool physical literacy system: motivation and Confidence: 25.68%, Physical Competence: 25.38%, Knowledge and Understanding: 23.42%, and Physical Activity Participation: 25.52%.

**Figure 2:**
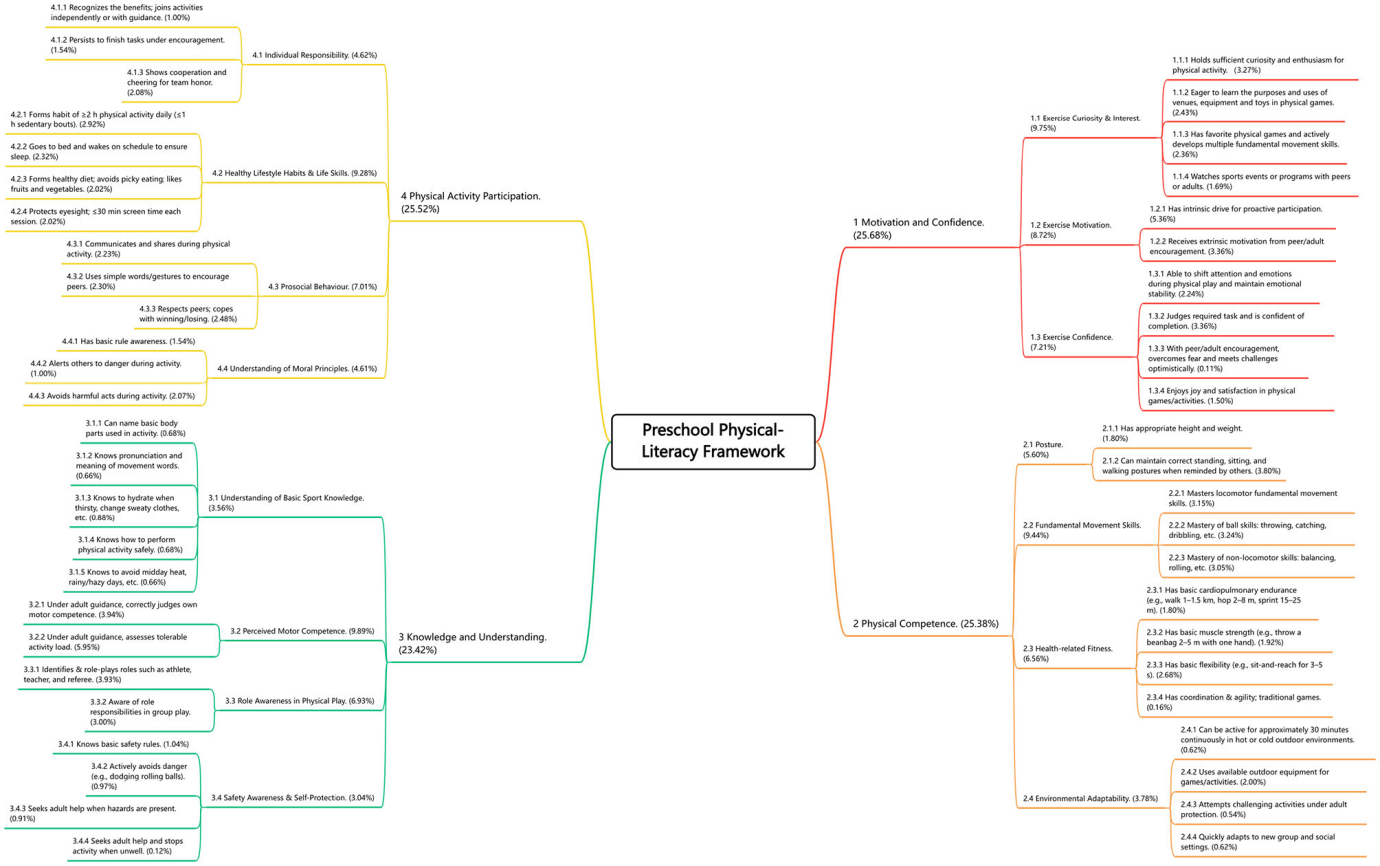
Final preschool physical literacy framework.

These results indicate that the four dimensions hold relatively equal importance within the overall framework. The weights of all dimensions, first-level indicators, and second-level indicators are detailed in Figure 2 and Appendix Table 3. In addition, the AHP calculation details, including the pairwise comparison matrices for dimensions and first-level indicators, are provided in Appendix Tables 4–8.

## 4 Discussion

In light of the current state of health promotion for preschool children in China and the national policy context, this study represents the first attempt to construct a comprehensive preschool PL framework. The resulting system includes four core dimensions—motivation and confidence, physical competence, knowledge and understanding, and physical activity participation—along with 15 first-level indicators and 49 second-level indicators, each with corresponding weight coefficients.

It is essential to cultivate children's interest in movement from an early age to help them develop sufficient motivation and confidence through enjoyable physical activity experiences. These foundational traits play a vital role in supporting the lifelong development of PL. According to the weight analysis, motivation and confidence emerged as the most critical dimension in the initiation and cultivation of PL during the preschool years. Experts also ranked this dimension as the most important across the entire system, underscoring its role as the driving force for preschoolers' PL development.

The dimension of knowledge and understanding reflects the external manifestation of a child's “physical culture” during early childhood. Experts generally agreed that, compared to the other three dimensions, this aspect carries relatively less weight at the preschool stage. This may be attributed to the fact that cognitive abilities in preschool children are still in the early stages of development, which further supports the rationality of its lower assigned weight in the framework.

Nevertheless, fostering this dimension remains important. Stakeholders such as parents and teachers should be actively involved in guiding and supporting children to acquire fundamental knowledge related to physical activity. Through this process, children can enhance their understanding of sports and movement, thereby gaining an early foundation for becoming physically literate individuals in the future.

This dimension comprises four key components: understanding of Basic Exercise Knowledge, Perceived Motor Competence, Role Awareness in Physical Play, and Safety Awareness & Self-Protection.

Understanding exercise knowledge forms the cognitive basis for engaging in physical activity. Without it, children's participation may become mechanical and repetitive, lacking enjoyment and sustainability. As Whitehead emphasized, relevant knowledge enables individuals to recognize the value and benefits of physical activity (19), fostering positive attitudes and intentions, and motivating sustained engagement in healthy behaviors. Empirical studies have also confirmed a positive correlation between physical activity knowledge and participation enthusiasm (20). Children with greater knowledge and more favorable attitudes toward movement are more likely to be actively involved in physical activities (21). Thus, cognition not only affirms the importance of movement but also serves as an internal motivator that underpins the development of PL.

Preschool years represent a critical window for the development of fundamental motor skills, making the dimension of physical competence especially significant in fostering PL during this period. This aligns with the developmental theory of sensitive periods, which suggests that early childhood is the optimal time for cultivating foundational movement abilities. According to the weighting results, physical competence is one of the most heavily weighted dimensions in the framework, highlighting its centrality to preschool PL development.

Parents, educators, and other stakeholders should fully recognize the developmental importance of physical competence and seize this critical period to enhance children's motor skill acquisition. From a developmental perspective, PL in early childhood must be grounded in regular and structured physical activity. Thus, physical competence serves as a foundational driver of PL acquisition and progress.

The dimension of physical activity participation ranked just behind physical competence in terms of importance, reflecting its crucial role within the preschool PL framework. Participation in physical activities serves both as a fundamental pathway for and a practical guarantee of developing PL in young children.

Therefore, parents, teachers, and other stakeholders should intentionally create diverse opportunities—both in time and space—for preschoolers to engage in structured and unstructured physical activities. Through rich and varied physical play, children can enhance their physical abilities, strengthen their motivation and confidence, and deepen their understanding of movement and sports. These experiences collectively support the initiation and cultivation of PL in early childhood.

Behavior related to physical activity comprises two key components: physical activity behaviors and risk prevention behaviors. These behaviors reflect the external manifestation and integrated effect of one's knowledge and physical competence. As such, they serve as a key mediator in the development of PL. Notably, this study identified physical activity–related behavior as the most heavily weighted domain in the evaluation framework, underscoring its central importance.

Existing research confirms that all forms of physical activity, regardless of intensity, are beneficial to health (22). To capture this comprehensively, the present framework includes light physical activity, moderate-to-vigorous physical activity, sedentary behavior, and sleep—collectively known as 24-h movement behaviors (23). However, previous PL models have primarily focused on MVPA and sedentary behavior, often overlooking LPA and sleep (24). It is important to recognize that even if a child possesses strong knowledge, skills, and attitudes, as well as high physical competence, their level of PL will remain low if they do not actively participate in physical activity (25).

In addition, risk prevention behavior plays a vital role in ensuring the safety and sustainability of physical activity participation by helping to prevent injuries that might otherwise hinder ongoing engagement.

Physical competence refers to the motor skills and physical fitness an individual acquires through participation in physical activities. It represents both a goal and a pathway for the development of PL. On one hand, engaging in physical activity promotes the enhancement of physical competence; on the other hand, improved physical competence can in turn boost a child's motivation and willingness to participate in physical activity (26).

Physical competence is closely linked to physical health and can be divided into six subdomains: body morphology and composition, cardiorespiratory endurance, muscular strength, flexibility, balance, and motor skills. Children who acquire a broad repertoire of motor skills are better equipped to adapt to a variety of physical environments. This adaptability enhances their confidence and initiative in engaging in physical activity and allows them to experience joy and satisfaction through movement (27).

From this perspective, physical competence not only reflects functional capabilities but also forms the physiological basis for health improvements driven by physical activity. In fact, physical competence can be viewed as both a foundation and a prerequisite for health status (28). Existing studies have also demonstrated a positive correlation between physical competence and physical activity participation: individuals with higher levels of competence tend to engage in physical activity more frequently and for longer durations (29). Therefore, physical competence is a crucial prerequisite for enhancing overall PL.

In summary, the four core dimensions—motivation and confidence, physical competence, knowledge and understanding, and physical activity participation—constitute the first-level indicators of the preschool PL framework. These dimensions are designed to complement and reinforce one another, forming an integrated and holistic system. Collectively, they serve as the four foundational pillars of PL for preschool children, each representing a fundamental aspect of PL development: the starting point, the pathway, the core, and the ultimate goal.

Preschoolers who demonstrate ideal levels of PL—characterized by well-developed movement knowledge, positive attitudes and intentions, consistent and sufficient physical activity, minimal sedentary behavior, adequate sleep, effective injury prevention strategies, good physical fitness, and diverse motor skills—are more likely to maintain an active and healthy lifestyle.

The preschool PL assessment system developed in this study provides a holistic tool for understanding and promoting physical activity behaviors in early childhood. It holds practical value in addressing insufficient physical activity and related health risks.

First, this framework enables comprehensive evaluation of children's responsiveness to physical activity and health promotion, offering a useful reference for public health practitioners. Second, it can help identify barriers to healthy behaviors and guide targeted interventions to foster active lifestyles.

Unlike existing PL tools, many of which are designed for school-aged children or specific contexts (30), this is the first framework in China tailored to preschoolers from a public health perspective. Notably, it introduces the dimension of “risk prevention behaviors” for the first time and includes a comprehensive view of 24-h movement behaviors—covering light activity, MVPA, sedentary time, and sleep—which are often underrepresented in similar tools.

To enhance its theoretical robustness and internal coherence, the framework is grounded in Whitehead's foundational definition of physical literacy and draws on behavior change theory and embodied cognition theory (19). This theoretical integration strengthens the conceptual foundation of the framework and clarifies the interrelationships among its core dimensions. Although developed within China's unique sociocultural and policy context, the methodological approach adopted in this study may offer valuable insights for other countries seeking to design PL tools for young children.

Finally, in response to ongoing international discussions about the inclusion of a “social dimension” in PL frameworks, this study incorporates relevant social indicators into the “physical activity participation” domain. This design choice aims to support the early development of social adaptability through participation in group-based physical activities, aligning the framework with both global trends and early childhood developmental needs.

To support its practical application, this framework can be operationalized through observation-based assessment tools administered by trained early childhood educators, health professionals, or physical activity specialists in community or preschool settings. Future work should focus on developing and validating a corresponding assessment scale and accompanying user guide to standardize its application across different regions and evaluator backgrounds.

Despite these strengths, several limitations should be acknowledged.

First, the distribution of experts across different research fields was somewhat uneven, which may have introduced bias in the weight assignments of certain indicators within the PL framework.

Second, as the framework was developed within China's specific context, comparisons with other countries—such as Australia—should be made cautiously. However, as Shearer et al. noted, PL frameworks are inherently context-specific and must align with local values and needs (31). Therefore, this localization can also be considered a methodological strength of our study.

Third, the study utilized the Analytic Hierarchy Process (AHP), a subjective weighting method, which may have introduced some degree of bias in the computed weights of the indicators.

Fourth, while the two-round Delphi design balances reliability and response burden, certain indicators (e.g., with consensus rates slightly below 80%) were retained based on expert reasoning and theoretical relevance, which may raise concerns about subjective interpretation. Clear documentation of these decisions was therefore essential.

Finally, this study did not proceed to empirical validation of the framework, which limits its immediate generalizability. Future research should test the reliability and validity of the indicators through field trials and psychometric analysis.

## 5 Conclusions

This study developed the first localized framework of PL for Chinese preschool children using a modified Delphi method and AHP. The framework, consisting of four core dimensions with weighted indicators, addresses a critical gap by contextualizing PL in early childhood within China's sociocultural setting. It provides a solid foundation for assessment, curriculum design, and health promotion efforts. Future research should focus on developing validated measurement tools based on this framework, and exploring its associations with physical activity, development, and health outcomes. These efforts will advance both the theoretical understanding and practical application of PL in early childhood.

## Data Availability

The data analyzed in this study is subject to the following licenses/restrictions: the raw data supporting the conclusions of this article will be made available by the authors, without undue reservation. Requests to access these datasets should be directed to Xiaojuan Tao, xjtao@suda.edu.cn.
