# Supplementary material for: Development of a physical literacy assessment framework for Chinese preschool children: a Delphi–AHP approach
Source: Front Public Health. 2025 Aug 19;13:1650793. doi: 10.3389/fpubh.2025.1650793 (PMC12401909; doi:10.3389/fpubh.2025.1650793)
Supplement: Supplementary file 1 [file Table_1.docx]

**Appendix Table 1. List of Consultation Experts**

| **No.** | **Name** | **Gender** | **Professional Job Title ^b^** | **Education** | **Area of Expertise ^c^** | **Affiliation** | **YOW ^a^** |
| --- | --- | --- | --- | --- | --- | --- | --- |
| 1 | Ren * | Male | Professor | PhD | Physical Literacy | Beijing Sport University | 49 |
| 2 | Shen ** | Male | Professor | PhD | Physical Literacy | The Chinese University of Hong Kong | 45 |
| 3 | Lin ** | Female | Professor | PhD | Physical Literacy | National Taiwan Normal University | 25 |
| 4 | Chen ** | Male | Professor | PhD | Physical Literacy | Victoria University (Australia) | 5 |
| 5 | Yin ** | Male | Associate Professor | PhD | Physical Literacy | East China Normal University | 7 |
| 6 | Ning * | Male | Associate Professor | PhD | Preschool Physical Education | Shaanxi Normal University | 18 |
| 7 | Zhao ** ^d^ | Female | Professor | PhD | Physical Literacy | Shandong Sport University | 8 |
| 8 | Gao ** ^d^ | Male | Associate Professor | PhD | Physical Literacy | Tianshui Normal University | 13 |
| 9 | Pan ** | Male | Professor | Bachelor | School Physical Education | Yangzhou University | 43 |
| 10 | Tang * | Male | Professor | PhD | School Physical Education | Shanghai University of Sport | 20 |
| 11 | Zhao ** | Male | Professor | PhD | School Physical Education | Wuhan Sports University | 18 |
| 12 | Ma ** ^d^ | Male | Associate Professor | PhD | School Physical Education | East China Normal University | 7 |
| 13 | Zeng * | Male | Associate Professor | PhD | School Physical Education | Hubei University of Arts and Science | 26 |
| 14 | Zhang * | Male | Associate Professor | PhD | School Physical Education | East China Normal University | 4 |
| 15 | Wang ** | Male | Associate Professor | Bachelor | Philosophy of Physical Education | Zhenjiang Education Center | 20 |
| 16 | Zhuang * | Male | Professor | Master | School Physical Education | Guangdong Provincial Department of Education | 43 |
| 17 | Shi ** | Female | Associate Professor | Master | School Physical Education | Nanjing Sport Institute | 13 |
| 18 | Zhao ** | Male | Professor | PhD | School Physical Education | Wenzhou University | 46 |
| 19 | Zhang * | Female | Professor | PhD | Preschool Physical Education | Zhejiang Normal University | 30 |
| 20 | Ma * | Female | Professor | PhD | Preschool Physical Education | Shanghai Normal University | 26 |
| 21 | Li ** | Female | Associate Professor | PhD | Preschool Physical Education | University of Macau | 15 |
| 22 | Guo *hua | Female | Professor | PhD | Preschool Physical Education | Changsha Normal University | 29 |
| 23 | Liu * ^d^ | Female | Associate Professor | PhD | Preschool Education | East China Normal University | 6 |
| 24 | Hou ** | Female | Teaching Assistant | Master | Preschool Instruction | Yantian District Educational Institute | 1 |
| 25 | Wu ** | Female | Associate Professor | Bachelor | Preschool Education | Taiyuan Dama Preschool | 26 |
| 26 | Wang ** | Female | Associate Professor | Bachelor | Preschool Physical Education | Yantai Liujia Preschool | 6 |
| 27 | Bai ** | Female | Teaching Assistant | Master | Preschool Education | Penglai Preschool | 8 |
| 28 | Chen * | Male | Associate Professor | Bachelor | Preschool Education | Penglai Preschool | 26 |
| 29 | Men ** ^d^ | Female | Associate Professor | Bachelor | Preschool Instruction | Zhongmei Beike EdTech | 7 |
| 30 | Fan ** ^d^ | Male | Associate Professor | Bachelor | Preschool Physical Education | Affiliated Zizhu Kindergarten of East China Normal University | 9 |
| 31 | Xiang ** | Male | Associate Professor | Bachelor | Preschool Instruction | Shenzhen Liyuan Education Group | 1 |
| 32 | Zhang ** | Male | Teaching Assistant | Master | Preschool Instruction | Capital Normal University Preschool | 3 |
| 33 | Liu ** | Female | Assistant Professor | Bachelor | Preschool Instruction | Dalian Hi-Tech Preschool | 10 |
| 34 | Chi ** | Female | Teaching Assistant | Master | Preschool Instruction | Yantai Liujia Preschool | 1 |
| 35 | Li ** ^d^ | Female | Teaching Assistant | Master | Preschool Instruction | Gefei Sports Co., Ltd. | 5 |
| 36 | Wang * ^d^ | Male | Associate Professor | Bachelor | Preschool Childcare | Yantai Liujia Preschool | 23 |
| 37 | Li * ^d^ | Male | Teaching Assistant | PhD | Preschool Childcare | Shanghai University of Sport | 1 |
| 38 | Ye ** ^d^ | Male | Assistant Professor | Bachelor | Preschool Childcare | Nanjing Sport Institute | 10 |
| 39 | Gong * ^d^ | Male | Teaching Assistant | Bachelor | Preschool Childcare | Shapingba Experimental School | 12 |
| 40 | Chang **^d^ | Male | Assistant Professor | Master | Preschool Childcare | Jiangsu University of Science and Technology (Zhangjiagang) | 11 |

a. YOW: years of work, calculated as of September 1, 2024.

b. All professional job titles were standardized based on the commonly used academic rank system in China: Teaching Assistant, Assistant Professor, Associate Professor, and Professor.

c. In the “Area of Expertise” category, “Preschool Education” refers to scholars focused on preschool education theory; “Preschool Physical Education” refers to experts in physical education working in early childhood contexts; and “Preschool Instruction” refers to frontline practitioners such as preschool PE teachers or coaches at early childhood sports clubs.

d. indicates that the expert is a parent of a preschool-aged child.

**Appendix Table 2. Full Translation of Indicator Revisions after Round 1 Expert Consultation**

| **No.** | **Original Indicator** | **Revision Action** | **Revised Indicator** |
| --- | --- | --- | --- |
| 1 | 4. Lifelong Physical Activity Participation | Modified | 4. Physical Activity Participation |
| 2 | 2.1 Posture | Added per Guidelines | — |
| 3 | 2.2 Physical Coordination | Deleted | — |
| 4 | 2.4 Environmental Adaptability and Risk-taking | Modified | 2.4 Environmental Adaptability |
| 5 | 3.1 Knowledge of Physical Activity | Modified | 3.1 Understanding of Basic Sport Knowledge |
| 6 | 3.5 Understanding of Moral Principles | Re-assigned | Moved to 4.4 |
| 7 | 4.2 Lifelong Healthy Lifestyle | Modified | 4.2 Healthy Lifestyle Habits & Life Skills |
| 8 | 4.4 Participation in Emerging Activities | Deleted | — |
| 9 | 1.1.1 Shows curiosity, novelty-seeking and enthusiasm for physical games/activities; tries and participates proactively. | Modified | 1.1.1 Holds sufficient curiosity and enthusiasm for physical activity. |
| 10 | 1.1.2 Interested in the use of equipment and facilities. | Modified | 1.1.2 Eager to learn the purposes and uses of venues, equipment and toys in physical games. |
| 11 | 1.1.3 Has favorite sports/games and is committed to 1–2 regularly. | Modified | 1.1.3 Has favorite physical games and actively develops multiple fundamental movement skills. |
| 12 | 1.1.4 Appreciates specific sports, follows events or athletes, cheers for success. | Modified | 1.1.4 Watches sports events or programs with peers or adults. |
| 13 | 1.2.1 Has intrinsic drive to participate actively in physical games/activities. | Modified | 1.2.1 Has intrinsic drive for proactive participation. |
| 14 | 1.2.2 Gets extrinsic motivation from environment to participate. | Modified | 1.2.2 Receives extrinsic motivation from peer/adult encouragement. |
| 15 | 1.3.1 Able to shift attention and emotions during physical play and maintain emotional stability. | Added per Guidelines | — |
| 16 | 1.3.1 Holds a winning belief in completing tasks. | Modified | 1.3.1 Judges the required task and is confident of completion. |
| 17 | 1.3.2 Can overcome fear and anxiety in physical challenges. | Modified | 1.3.2 With peer/adult encouragement, overcomes fear and meets challenges optimistically. |
| 18 | 1.3.3 Enjoys pleasure and satisfaction after completion. | Modified | 1.3.3 Enjoys joy and satisfaction in physical games/activities. |
| 19 | 2.1.1 Has appropriate height and weight. | Added per Guidelines | — |
| 20 | 2.1.2 Can maintain correct standing, sitting, and walking postures when reminded by others. | Added per Guidelines | — |
| 21 | 2.1.1 Masters locomotor fundamental movement skills. | Modified | 2.1.1 Basically masters locomotor FMS. |
| 22 | 2.3.1 Demonstrates cardiorespiratory endurance. | Modified | 2.3.1 Has basic cardiopulmonary endurance (e.g., walking 1–1.5 km, hopping 2–8 m, sprinting 15–25 m). |
| 23 | 2.3.2 Shows muscular strength through duration of activity. | Modified | 2.3.2 Has basic muscle strength (e.g., throwing a beanbag 2–5 m with one hand). |
| 24 | 2.3.3 Possesses flexibility in shoulders, hips, and trunk. | Modified | 2.3.3 Has basic flexibility (e.g., performing sit-and-reach for 3–5 s). |
| 25 | 2.3.4 Has coordination & agility; traditional games. | Added per Guidelines | — |
| 26 | 2.4.1 Adapts to outdoor environments and varied conditions. | Modified | 2.4.1 Can be active for approximately 30 minutes continuously in hot or cold outdoor environments. |
| 27 | 2.4.2 Uses available equipment for diverse outdoor activities. | Modified | 2.4.2 Uses available outdoor equipment for games/activities. |
| 28 | 2.4.3 Willingly engages in adventurous physical tasks. | Modified | 2.4.3 Attempts challenging activities under adult protection. |
| 29 | 2.4.4 Quickly adapts to new group and social settings. | Added per Guidelines | — |
| 30 | 3.1.1 Knows basic body structure & own status. | Modified | 3.1.1 Can name basic body parts used in activity. |
| 31 | 3.1.2 Knows pronunciation & writing of movement vocab. | Modified | 3.1.2 Knows pronunciation and meaning of movement words. |
| 32 | 3.1.3 Knows the benefits of physical activity. | Modified | 3.1.3 Knows to hydrate when thirsty, change sweaty clothes, etc. |
| 33 | 3.1.4 Knows how to perform physical activity safely. | Deleted | — |
| 34 | 3.1.5 Aware of appropriate timing for physical activity. | Modified | 3.1.5 Knows to avoid midday heat, rainy/hazy days, etc.. |
| 35 | 3.2.1 Sensitive to own bodily reactions during activity. | Re-assigned & Modified | Moved up to 3.1, replaces 3.1.3 (text merged) |
| 36 | 3.2.2 Accurately judges personal motor and activity limits. | Modified | 3.2.2 Under adult guidance, correctly judges own motor competence. |
| 37 | 3.2.3 Recognizes discomfort signs like cramps or pain. | Modified | 3.2.3 Under adult guidance, assesses tolerable activity load. |
| 38 | 3.3.1 Understands and imitates social roles in games. | Modified | 3.3.1 Identifies & role-plays roles such as athlete, teacher, and referee. |
| 39 | 3.4.1 Knows basic safety rules. | Added per Guidelines | — |
| 40 | 3.4.1 Practices basic injury prevention strategies. | Modified | 3.4.1 Actively avoids danger (e.g., dodging rolling balls). |
| 41 | 3.4.2 Identifies and avoids hazards in the environment. | Modified | 3.4.2 Seeks adult help when hazards are present. |
| 42 | 3.4.3 Participates cautiously; stops when unwell. | Modified | 3.4.3 Seeks adult help and stops activity when unwell. |
| 43 | 3.5.1 Has basic rule awareness. | Re-assigned | Renumbered 4.4.1 |
| 44 | 3.5.2 Follows basic social rules in daily life. | Deleted | — |
| 45 | 3.5.3 Alerts others to danger during activity. | Re-assigned | Renumbered 4.4.2 |
| 46 | 3.5.4 Avoids harmful acts during activity. | Re-assigned | Renumbered 4.4.3 |
| 47 | 4.1.1 Views exercise as lifelong responsibility. | Modified | 4.1.1 Recognizes the benefits; joins activities independently or with guidance. |
| 48 | 4.1.2 Shows perseverance and courage. | Modified | 4.1.2 Persists to finish tasks under encouragement. |
| 49 | 4.1.3 Contributes for collective honor. | Modified | 4.1.3 Shows cooperation and cheering for team honor. |
| 50 | 4.2.1 Forms proactive exercise habit (60 min+60 min). | Modified | 4.2.1 Forms habit of ≥2 h physical activity daily (≤1 h sedentary bouts). |
| 51 | 4.2.2 Maintains good sleep habits and rest. | Modified | 4.2.2 Goes to bed and wakes on schedule to ensure sleep. |
| 52 | 4.2.3 Forms healthy dietary habits. | Modified | 4.2.3 Forms healthy diet; avoids picky eating; likes fruits and vegetables. |
| 53 | 4.2.4 Protects eyesight; ≤30 min screen time each session. | Added per Guidelines | — |
| 54 | 4.3.2 Helps and comforts peers in difficulty. | Modified | 4.3.2 Uses simple words/gestures to encourage peers. |
| 55 | 4.3.3 Respects peers; copes with winning/losing. | Added (Child behavioral psych) | — |

a. In the indicator text, physical activity is an independent noun referring to all bodily movement other than “physical games”.

b. “Guidelines” refers to the Guidelines for Learning and Development of Children Aged 3–6 issued by the Ministry of Education of China.

**Appendix Table 3. Weight Table of Indicators in the Preschool Physical-Literacy Framework**

| **No.** | **Dimension Indicator** | **Wt (%)** | **First-level Indicator** | **Wt (%)** | **Second-level Indicator** | **Wt (%)** |
| --- | --- | --- | --- | --- | --- | --- |
| 1 | 1 Motivation and Confidence | 25.68 | 1.1 Exercise Curiosity & Interest | 9.75 | 1.1.1 Holds sufficient curiosity and enthusiasm for physical activity. | 3.27 |
| 2 |  |  |  |  | 1.1.2 Eager to learn the purposes and uses of venues, equipment and toys in physical games. | 2.43 |
| 3 |  |  |  |  | 1.1.3 Has favorite physical games and actively develops multiple fundamental movement skills. | 2.36 |
| 4 |  |  |  |  | 1.1.4 Watches sports events or programs with peers or adults. | 1.69 |
| 5 |  |  | 1.2 Exercise Motivation | 8.72 | 1.2.1 Has intrinsic drive for proactive participation. | 5.36 |
| 6 |  |  |  |  | 1.2.2 Receives extrinsic motivation from peer/adult encouragement. | 3.36 |
| 7 |  |  | 1.3 Exercise Confidence | 7.21 | 1.3.1 Able to shift attention and emotions during physical play and maintain emotional stability. | 2.24 |
| 8 |  |  |  |  | 1.3.2 Judges required task and is confident of completion. | 3.36 |
| 9 |  |  |  |  | 1.3.3 With peer/adult encouragement, overcomes fear and meets challenges optimistically. | 0.11 |
| 10 |  |  |  |  | 1.3.4 Enjoys joy and satisfaction in physical games/activities. | 1.50 |
| 11 | 2 Physical Competence | 25.38 | 2.1 Posture | 5.6 | 2.1.1 Has appropriate height and weight. | 1.8 |
| 12 |  |  |  |  | 2.1.2 Can maintain correct standing, sitting, and walking postures when reminded by others. | 3.80 |
| 13 |  |  | 2.2 Fundamental Movement Skills | 9.44 | 2.2.1 Masters locomotor fundamental movement skills. | 3.15 |
| 14 |  |  |  |  | 2.2.2 Mastery of ball skills: throwing, catching, dribbling, etc. | 3.24 |
| 15 |  |  |  |  | 2.2.3 Mastery of non-locomotor skills: balancing, rolling, etc. | 3.05 |
| 16 |  |  | 2.3 Health-related Fitness | 6.56 | 2.3.1 Has basic cardiopulmonary endurance (e.g., walk 1–1.5 km, hop 2–8 m, sprint 15–25 m). | 1.80 |
| 17 |  |  |  |  | 2.3.2 Has basic muscle strength (e.g., throw a beanbag 2–5 m with one hand). | 1.92 |
| 18 |  |  |  |  | 2.3.3 Has basic flexibility (e.g., sit-and-reach for 3–5 s). | 2.68 |
| 19 |  |  |  |  | 2.3.4 Has coordination & agility; traditional games. | 0.16 |
| 20 |  |  | 2.4 Environmental Adaptability | 3.78 | 2.4.1 Can be active for approximately 30 minutes continuously in hot or cold outdoor environments. | 0.62 |
| 21 |  |  |  |  | 2.4.2 Uses available outdoor equipment for games/activities. | 2.00 |
| 22 |  |  |  |  | 2.4.3 Attempts challenging activities under adult protection. | 0.54 |
| 23 |  |  |  |  | 2.4.4 Quickly adapts to new group and social settings. | 0.62 |
| 24 | 3 Knowledge and Understanding | 23.42 | 3.1 Understanding of Basic Sport Knowledge | 3.56 | 3.1.1 Can name basic body parts used in activity. | 0.68 |
| 25 |  |  |  |  | 3.1.2 Knows pronunciation and meaning of movement words. | 0.66 |
| 26 |  |  |  |  | 3.1.3 Knows to hydrate when thirsty, change sweaty clothes, etc. | 0.88 |
| 27 |  |  |  |  | 3.1.4 Knows how to perform physical activity safely. | 0.68 |
| 28 |  |  |  |  | 3.1.5 Knows to avoid midday heat, rainy/hazy days, etc. | 0.66 |
| 29 |  |  | 3.2 Perceived Motor Competence | 9.89 | 3.2.1 Under adult guidance, correctly judges own motor competence. | 3.94 |
| 30 |  |  |  |  | 3.2.2 Under adult guidance, assesses tolerable activity load. | 5.95 |
| 31 |  |  | 3.3 Role Awareness in Physical Play | 6.93 | 3.3.1 Identifies & role-plays roles such as athlete, teacher, and referee. | 3.93 |
| 32 |  |  |  |  | 3.3.2 Aware of role responsibilities in group play. | 3.00 |
| 33 |  |  | 3.4 Safety Awareness & Self-Protection | 3.04 | 3.4.1Knows basic safety rules. | 1.04 |
| 34 |  |  |  |  | 3.4.2 Actively avoids danger (e.g., dodging rolling balls). | 0.97 |
| 35 |  |  |  |  | 3.4.3 Seeks adult help when hazards are present. | 0.91 |
| 36 |  |  |  |  | 3.4.4 Seeks adult help and stops activity when unwell. | 0.12 |
| 37 | 4 Physical Activity Participation | 25.52 | 4.1 Individual Responsibility | 4.62 | 4.1.1 Recognizes the benefits; joins activities independently or with guidance. | 1.00 |
| 38 |  |  |  |  | 4.1.2 Persists to finish tasks under encouragement. | 1.54 |
| 39 |  |  |  |  | 4.1.3 Shows cooperation and cheering for team honor. | 2.08 |
| 40 |  |  | 4.2 Healthy Lifestyle Habits & Life Skills | 9.28 | 4.2.1 Forms habit of ≥2 h physical activity daily (≤1 h sedentary bouts). | 2.92 |
| 41 |  |  |  |  | 4.2.2 Goes to bed and wakes on schedule to ensure sleep. | 2.32 |
| 42 |  |  |  |  | 4.2.3 Forms healthy diet; avoids picky eating; likes fruits and vegetables. | 2.02 |
| 43 |  |  |  |  | 4.2.4 Protects eyesight; ≤30 min screen time each session. | 2.02 |
| 44 |  |  | 4.3 Prosocial Behaviour | 7.01 | 4.3.1Communicates and shares during physical activity. | 2.23 |
| 45 |  |  |  |  | 4.3.2 Uses simple words/gestures to encourage peers. | 2.3 |
| 46 |  |  |  |  | 4.3.3 Respects peers; copes with winning/losing. | 2.48 |
| 47 |  |  | 4.4 Understanding of Moral Principles | 4.61 | 4.4.1 Has basic rule awareness. | 1.54 |
| 48 |  |  |  |  | 4.4.2 Alerts others to danger during activity. | 1.00 |
| 49 |  |  |  |  | 4.4.3 Avoids harmful acts during activity. | 2.07 |

**Appendix Table 4. Pairwise Comparison Matrix for Dimensions of the Preschool PL Framework**

|  | **Motivation and Confidence** | **Physical Competence** | **Knowledge & Understanding** | **Physical Activity Participation** | **Mean** |
| --- | --- | --- | --- | --- | --- |
| **Motivation and Confidence** | 1.00 | 1.05 | 2.53 | 1.01 | 3.00 |
| **Physical Competence** | 0.95 | 1.00 | 2.41 | 0.96 | 2.85 |
| **Knowledge & Understanding** | 0.40 | 0.42 | 1.00 | 0.40 | 1.19 |
| **Physical Activity Participation** | 0.99 | 1.04 | 2.50 | 1.00 | 2.96 |

**Appendix Table 5. Pairwise Comparison Matrix for the First-Level Indicators of “Motivation and Confidence” Dimension**

|  | **Exercise Curiosity & Interest** | **Exercise Motivation** | **Exercise Confidence** | **Mean** |
| --- | --- | --- | --- | --- |
| **Exercise Curiosity & Interest** | 1.00 | 1.37 | 1.59 | 3.82 |
| **Exercise Motivation** | 0.73 | 1.00 | 1.15 | 2.78 |
| **Exercise Confidence** | 0.63 | 0.87 | 1.00 | 2.41 |

**Appendix Table 6. Pairwise Comparison Matrix for the First-Level Indicators of “Physical Competence” Dimension**

|  | **Posture** | **Fundamental Movement Skills** | **Health-related Fitness** | **Environmental Adaptability** | **Mean** |
| --- | --- | --- | --- | --- | --- |
| **Posture** | 1.00 | 0.54 | 0.65 | 0.82 | 1.70 |
| **Fundamental Movement Skills** | 1.87 | 1.00 | 1.21 | 1.54 | 3.33 |
| **Health-related Fitness** | 1.54 | 0.83 | 1.00 | 1.27 | 2.44 |
| **Environmental Adaptability** | 1.22 | 0.65 | 0.79 | 1.00 | 2.52 |

**Appendix Table 7. Pairwise Comparison Matrix for the First-Level Indicators of “Knowledge & Understanding” Dimension**

|  | **Understanding of Basic Sport Knowledge** | **Perceived Motor Competence** | **Role Awareness in Physical Play** | **Safety Awareness & Self-Protection** | **Mean** |
| --- | --- | --- | --- | --- | --- |
| **Understanding of Basic Sport Knowledge** | 1.00 | 0.51 | 0.70 | 0.68 | 1.70 |
| **Perceived Motor Competence** | 1.96 | 1.00 | 1.36 | 1.32 | 3.33 |
| **Role Awareness in Physical Play** | 1.44 | 0.73 | 1.00 | 0.97 | 2.44 |
| **Safety Awareness & Self-Protection** | 1.48 | 0.76 | 1.03 | 1.00 | 2.52 |

**Appendix Table 8. Pairwise Comparison Matrix for the First-Level Indicators of “Physical Activity Participation” Dimension**

|  | **Individual Responsibility** | **Healthy Lifestyle Habits & Life Skills** | **Prosocial Behaviour** | **Understanding of Moral Principles** | **Mean** |
| --- | --- | --- | --- | --- | --- |
| Individual Responsibility | 1.00 | 0.60 | 0.85 | 1.02 | 2.07 |
| Healthy Lifestyle Habits & Life Skills | 1.68 | 1.00 | 1.42 | 1.71 | 3.48 |
| Prosocial Behaviour | 1.18 | 0.70 | 1.00 | 1.20 | 2.44 |
| Understanding of Moral Principles | 0.98 | 0.59 | 0.83 | 1.00 | 2.04 |
